# An *In Vitro* Comparison of Embolus Differentiation Techniques for Clinically Significant Macroemboli: Dual-Frequency Technique versus Frequency Modulation Method

**DOI:** 10.1016/j.ultrasmedbio.2014.06.003

**Published:** 2014-11

**Authors:** Caroline Banahan, Zach Rogerson, Clément Rousseau, Kumar V. Ramnarine, David H. Evans, Emma M.L. Chung

**Affiliations:** ∗ Medical Physics Department, University Hospitals of Leicester NHS Trust, Leicester, UK; † Department of Physics, University of Leicester, Leicester, UK; ‡ Department of Cardiovascular Sciences, University of Leicester, Leicester, UK

**Keywords:** Embolus detection, Transcranial Doppler ultrasound, Discrimination, Emboli

## Abstract

The ability to distinguish harmful solid cerebral emboli from gas bubbles intra-operatively has potential to direct interventions to reduce the risk of brain injury. In this *in vitro* study, two embolus discrimination techniques, dual-frequency (DF) and frequency modulation (FM) methods, are simultaneously compared to assess discrimination of potentially harmful large pieces of carotid plaque debris (0.5–1.55 mm) and thrombus-mimicking material (0.5–2 mm) from gas bubbles (0.01–2.5 mm). Detection of plaque and thrombus-mimic using the DF technique yielded disappointing results, with four out of five particles being misclassified (sensitivity: 18%; specificity: 89%). Although the FM method offered improved sensitivity, a higher number of false positives were observed (sensitivity: 72%; specificity: 50%). Optimum differentiation was achieved using the difference between peak embolus/blood ratio and mean embolus/blood ratio (sensitivity: 77%; specificity: 81%). We conclude that existing DF and FM techniques are unable to confidently distinguish large solid emboli from small gas bubbles (<50 μm).

## Introduction

Showers of solid and gaseous emboli can enter the cerebral circulation peri-operatively, putting patients at risk of neurologic injury (Braekken et al., 1998, Kilicaslan et al., 2006, Schmitz et al., 2003). The ability to size and characterize cerebral emboli intra-operatively has potential to improve the safety profile of these procedures by enabling clinicians to identify the causes of emboli, predict embolus composition and diagnose likely clinical sequelae. Two embolus discrimination techniques, based on transcranial Doppler (TCD) ultrasound, have reported some success in distinguishing between small solid emboli and microbubbles (<100 μm diameter): the dual-frequency (DF) technique (Russell and Brucher, 2002, Russell and Brucher, 2005) and the frequency modulation (FM) method (Girault et al., 2011b, Smith et al., 1997). However, few validation studies have specifically evaluated these techniques for detection and classification of large solid particles, which generate very similar Doppler embolic signals to gas bubbles. In this *in vitro* study the performance of DF and FM techniques is specifically assessed by focusing on the most clinically relevant, and technically challenging, scenario of distinguishing large solid particles from small bubbles.

The dual-frequency technique was developed more than a decade ago based on theoretical models revealing the frequency dependence of Doppler embolic signal intensity for emboli of differing composition (Moehring and Klepper 1994). In 2002, a commercial TCD machine was developed that featured a dual-frequency transducer to insonate emboli at 2.0 and 2.5 MHz. Identification of small differences in backscattered embolic signal intensity at these two frequencies theoretically provides a means of distinguishing solid emboli from gas bubbles. Initial experimental work was promising (Russell and Brucher, 2002, Russell and Brucher, 2005), but unfortunately, subsequent clinical testing found poor sensitivity and specificity, leading Markus and Punter (2005) to conclude that this method was not accurate enough for use in clinical or research studies. Despite these findings, several groups continue to publish clinical trials featuring DF embolus discrimination, and the DF technique is currently available in commercial TCD systems (Abu-Omar et al., 2004, Chen et al., 2006, Guerrieri Wolf et al., 2008, Maselli et al., 2006, Nagy-Balo et al., 2013).

The frequency modulation method was first proposed by Smith et al. (1997), who postulated that the frequency modulation observed in Doppler embolic signatures was due to displacement of the embolus as it crosses the sample volume. Smith et al. (1997) observed that gaseous emboli tended to be characterized by stronger variations in Doppler frequency (frequency modulation) than solid emboli and categorized Doppler FM signatures as three main types: Type I had no FM and were seen predominantly for solid emboli; type II exhibited a gradual increase in FM over the signal duration and were measured for both types of emboli; type III exhibited sudden frequency modulation and were purely from gaseous emboli. Curvature of the artery, non-axial and helical flow, harmonic generation caused by interaction of emboli with the beam, ultrasound radiation force and phase cancelation were some of the theories hypothesized to explain these signatures. Souchon et al. (2005) suggested that the high FM associated with gas bubbles was due to acoustic radiation forces (ARFs) exerted by the ultrasound beam, which induces an “extra acceleration” that alters the trajectory of gas bubbles in the flow. Souchon et al. (2005) performed an *in vitro* study that clearly reported the effect of ARFs on the trajectories of small bubbles, but, as the authors point out, one might expect to see less frequency modulation *in vivo*, particularly from bubbles crossing the edges of the sample volume. FM-based techniques therefore have potential to produce a high false positive rate as a result of misclassified bubbles.

Girault et al. (2011b) used theoretical models to test whether ARFs were responsible for sudden changes in Doppler frequency associated with gas bubbles. Their simulations indicated that gaseous bubbles experienced stronger ARF displacements than solid emboli of a similar size (1–100 μm). To test their predictions, Girault et al. (2011b) performed an *in vitro* experiment and found that microbubbles <100 μm in diameter (R_0_) were successfully differentiated from solid fat particles (100 < R_0_ < 300 μm), even in the presence of distortion of the ultrasound beam by the skull under steady flow conditions. However, from their theoretical models the authors concluded that for pulsatile (and more clinically realistic) flow, “the shape and value of the additional acceleration seems to be very complex and unpredictable” (Girault et al. 2011a). Therefore, additional complications associated with TCD measurements *in vivo*, such as pulsatile flow and complex flow distributions, may have potential to mask ARF-induced frequency modulation and limit the success of this technique.

The aim of the current *in vitro* study was to directly compare existing commercially available dual-frequency embolus discrimination (Doppler-Box, Compumedics Germany GmbH, Singen, Germany) with FM methods for distinguishing thrombus and plaque particles from bubbles. The effects of pulsatile and non-pulsatile flow were measured for thrombus-mimicking emboli to investigate whether pulsatile flow limits the accuracy of the FM method by introducing additional FM within the Doppler signal. Doppler embolic signal characteristics were also analyzed for each embolus, to determine whether a combination of these properties could help differentiate between solid and gaseous emboli.

## Methods

### In vitro setup

**Fig. 1:**
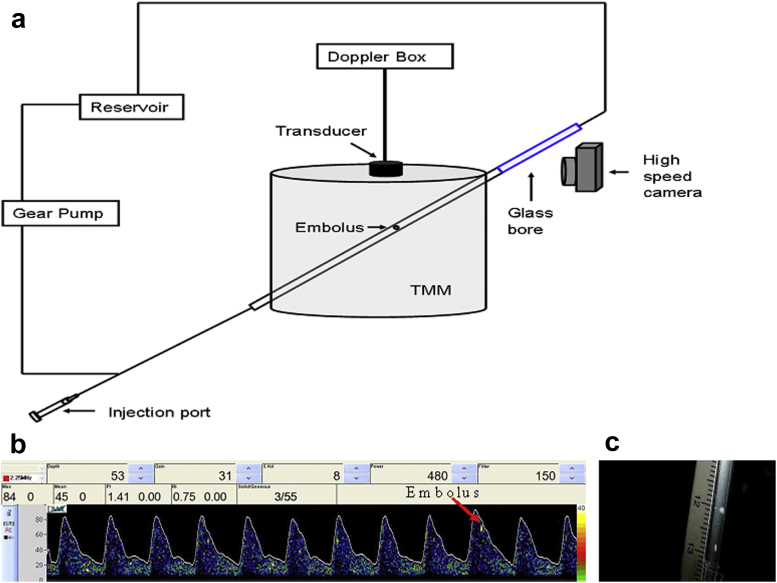
(a) A schematic of the flow phantom used for the *in vitro* experiment. Emboli were introduced into the phantom *via* an injection port. The embolic Doppler signal was recorded as the embolus passed through the Doppler sample volume and was subsequently imaged through a glass bore using a high-speed camera (600 fps). (b) Doppler waveform recorded from the phantom. For pulsatile flow, a pre-programmed wave function was used to circulate a blood-mimicking fluid (BMF) around the phantom. This had a pulsatile waveform to imitate a typical Doppler waveform in the middle cerebral artery at a frequency of 60 pulses per min, mean velocity ∼ 46 cm/s. An embolic signal appears as a high-intensity transient signal superimposed on the blood waveform in the spectrogram. (c) Pieces of tissue-mimicking material (TMM) imaged through the rectangular glass bore after leaving the Doppler sample volume.

To test the two embolus differentiation methods a flow phantom was developed (Fig. 1a). The circuit was constructed from C-flex tubing with 2.5 mm internal diameter and 0.8 mm wall thickness (Cole-Parmer, London, UK) (Hoskins and Ramnarine 2000). The container was filled with an agar-based tissue-mimicking material (TMM) (Browne et al., 2003, Ramnarine et al., 2001), which attenuates and backscatters the ultrasound beam similarly to tissue, and an injection port was placed in the circuit to allow introduction of emboli into the phantom. The tubing was placed in the container at an angle of 40 degrees to mimic the insonation geometry of the middle cerebral artery.

A programmable gear pump (Micropump Model 120-000-1100, Concord, CA, USA) was used to generate controllable pulsatile and non-pulsatile flow of a diluted, de-gassed blood-mimicking fluid (BMF) (Ramnarine et al. 1998). For pulsatile flow, a pre-programmed wave function was used to circulate BMF around the phantom with a pulsatile waveform imitating blood flow through the middle cerebral artery (MCA; Fig. 1b) at a frequency of 60 pulses/min. This generated a mean velocity of ∼46 cm/s in the circuit (average MCA velocity is 32–78 cm/s) (Lindegaard et al. 1987). For non-pulsatile flow, a mean velocity of 80 cm/s was used (average MCA velocity is 34–62 cm/s on cardiopulmonary bypass) (Endoh and Shimoji 1994).

Informed consent was obtained from patients undergoing carotid endarterectomy to enable us to utilize their excised carotid plaque for laboratory research. Consent was obtained in accordance with local ethics (07/Q2403/53, Nottingham 1, NRES committee, East Midlands) to retain and study their excised carotid plaque. The plaque sample was collected straight from the operating theater and stored in saline (0.9% NaCl). This excised plaque was cut into small pieces using a scalpel to form plaque emboli with diameters ranging between 0.5 and 1.55 mm. This size range was deemed appropriate to test how well both techniques perform in detecting clinically significant solid macroemboli that may pose a serious threat to the patient. Thrombus-mimicking particles of a similar size (0.5–2 mm) were made by cutting up a large piece of TMM. Fabrication of the TMM is described in Ramnarine et al. (2001), where the TMM is confirmed to have similar acoustic properties as thrombus and soft tissue (Duck 1990) (Table 1). All solid particles were sized under a calibrated microscope, and diameters were estimated to be accurate to ∼80 μm. Solid emboli were injected into the phantom in a 5 mL solution of BMF, with microbubbles generated by introducing small amounts of air into the flow rig and by the action of the gear pump. Care was taken to inject very small volumes of BMF with the solid emboli to ensure the blood flow waveform was not altered and to minimize the risk of introducing artifactual bubbles and artifacts. Bubble sizes were estimated using a validated algorithm developed by Banahan et al. (2012) based on the analysis of backscattered signal intensity. The algorithm is based on a theoretical model described by Lubbers and Van Den Berg (1977) and assumes a spherical embolus traveling in a blood-filled vessel with the ultrasound beam parallel to the imaged vessel; see Table 1 for the physical properties used in the model. The ratio of the backscattered intensity of an embolus in blood compared with the background blood signal is calculated and expressed as an embolus/blood ratio (EBR) in decibels, dB. By taking these measurements from the same sample volume, refraction and diffraction effects, along with attenuation of the beam through tissue, are effectively canceled out.

**Table 1:** Parameters used in the sizing algorithm described in Banahan et al. (2012) to estimate diameter of air bubbles traveling in a weakly diluted BMF solution. Acoustic properties of blood, tissue (Duck 1990) and tissue-mimicking material (Ramnarine et al. 1998) are also given

Modeling assumptions *in vitro*		
BMF & air	Density kg·m^−3^	Speed of sound m·s^−1^
BMF	1037	1547
Orgasol particles	1060	2380
Plasma (glycerol mixture)	1022	1550
Air	1.1387	353.3

Blood, tissue & TMM	Density kg·m^−3^	Speed of sound m·s^−1^

Blood	1055	1580
Tissue (average value)	1000–1070	1540
TMM	1054	1551
Orgasol assumptions	Diameter	5 μm
	Number density (δ_bmf_)	3.06 × 10^13^ m^−3^
	Cross section (σ_bmf_)	4.15 × 0^−20^ m^−2^
	Hematocrit (H)	0.002%
	Packing factor (W)	1
Insonating artery	Vessel radius (R)	1.25 mm
	SVL∗	10.44 mm

BMF = blood-mimicking fluid; TMM = tissue-mimicking material; SVL = sample volume length.

∗ SVL (sample volume length) calculated for 8 mm sample volume at an angle of 40 degrees.

Emboli were imaged after leaving the insonation volume through a 4 mm rectangular glass bore (S103, Composite Metal Services Ltd, Shipley, UK) using a high-speed, 600 fps camera (EX-F1 Exilim, Casio Computer Co. Ltd., Tokyo, Japan) (Fig. 1c). To ensure clear images of each embolus, the percentage of 5 μm orgasol scatterers in the BMF was reduced from 1.82% to 0.02%. This diluted suspension generated a Doppler background signal that was approximately 1/100 the strength of that of blood (Martin et al. 2009) but does not alter blood flow velocity measurements. The recorded videos of emboli were examined frame by frame using Quicktime Player (Apple Inc., Cupertino, CA, USA) to identify when a solid embolus had passed through the Doppler sample volume.

Doppler signals were obtained using a commercial TCD machine, equipped with a dual-frequency transducer with center frequency 2.25 MHz, operating at 2.0 and 2.5 MHz (Doppler-Box, DWL). TCD settings included a sample volume length of 8 mm and sample depth of 53 mm, at an angle of 40 degrees to the artificial vessel mimicking typical TCD settings for *in vivo* insonation of the middle cerebral artery.

Data were recorded in continuous mode with embolus detection and differentiation software enabled and using the manufacturer's default embolus detection threshold of 9 dB above the background blood signal. This threshold was used in previously published clinical research, so it was decided to test the software using this same value in the present study (Guerrieri Wolf et al., 2008, Nagy-Balo et al., 2013). This differentiation software generated a table containing the times of detected emboli, estimated embolus composition and EBR values, which were then imported into Excel (Microsoft, Redmond, WA, USA) for further analysis. The software rejects signals as artifacts if the signal appears at more than 1 depth simultaneously.

### Analysis

**Fig. 2:**
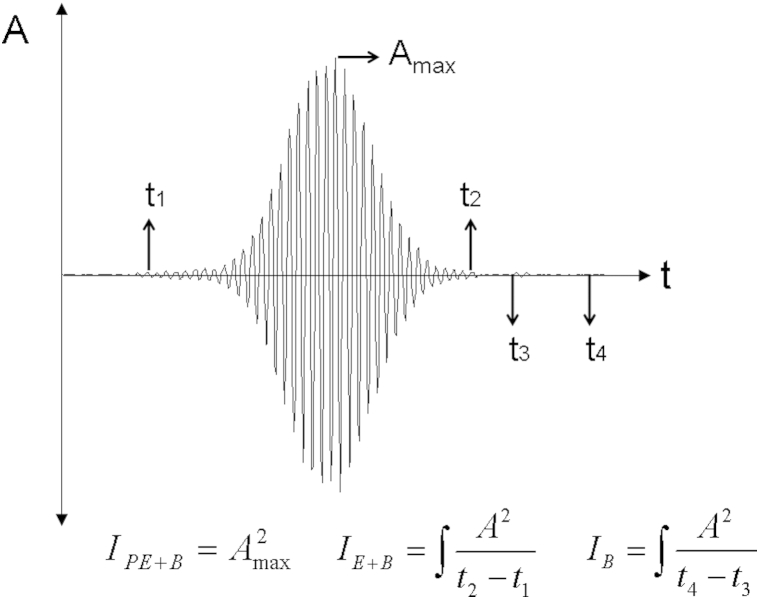
An example of an embolic signal. The abscissa represents time *(t)* and the ordinate amplitude *(A)*. The embolic signal duration is given by t_2_-t_1_ and the background signal is measured from t_3_ to t_4_. Calculation of the peak backscattered signal intensity is given by I_PE+B_, the average backscattered intensity, I_E+B_, and the backscattered signal intensity from the background blood (BMF) signal, I_B_.

The raw audio data from 2 MHz was available to export in bin file format from the machine’s software. This audio data was subsequently analyzed using in-house software developed in Matlab (Mathworks Inc., Natick, MA, USA). Embolic signals were located as peaks within the recording by using a detection threshold of 5 dB above the average background intensity calculated by averaging the signal over the entire recording. Note that this difference in detection threshold from the automatic software was necessary to successfully identify emboli within the recorded signal using the in-house software: The automatic software calculates the background average from either side of the embolic signal and not from averaging the background over the entire recording. Indeed where recordings contained several very high intensity peaks, a lower threshold of 3 dB was necessary to ensure all weaker emboli were detected within the data set. Two background windows were carefully selected either side of the embolus peak to ensure that no artifacts were present, and these were averaged before being integrated and normalized with respect to time. Signals were classified as artifacts if they appeared in both the recorded forward and reverse blood flow channel: Emboli should only appear in the forward channel as the blood flow is directed toward the ultrasound beam. The peak intensity of backscatter from the embolus, measured as a peak EBR (PEBR), was then determined using Equation 1:

(1) $$PEBR=10\;\log_{10}\left(\frac{I_{PE+B}}{I_{B}}\right)\text{dB}$$

where I_PE+B_ is the peak intensity of the embolic signal in blood (the maximum amplitude of the backscattered signal from the embolus in blood squared) and I_B_ is the intensity of the average background audio signal (the integrated average backscattered power of the background blood with respect to time) (Fig. 2). Theoretically, this equation corresponds to the ratio of the peak acoustic backscattered signal from the embolus to that of the surrounding blood. Ultrasound scattering theory predicts that gaseous emboli will backscatter ultrasound with much greater intensity than a solid embolus of the same size because of the larger acoustic impedance mismatch between air and blood. This makes PEBR a prime candidate for attempting to distinguish between gaseous and solid emboli.

**Fig. 3:**
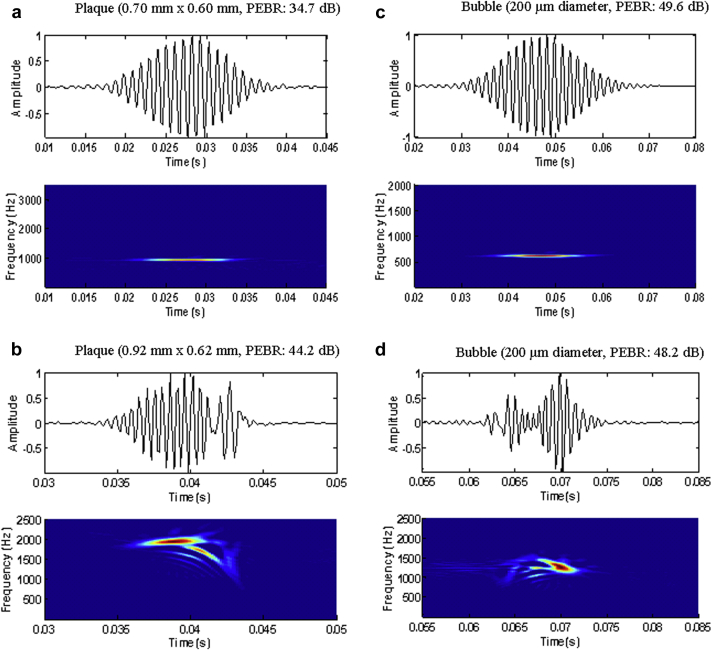
(a, b) Plaque and (c, d) bubble embolic signals. For each figure, the *top panel* is a time representation of the Doppler embolic signal and the *bottom panel* shows the time frequency representation (Wigner-Ville transform for improved time-frequency resolution). Doppler signals were recorded using a commercial TCD system (Doppler-Box, DWL). The system settings were PRF = 5 kHz, N = 25 cycles, f = 2.0 MHz, I_spta_ = 480 mW/cm^2^. The frequency modulation index for each Doppler signal was (a) 2, (b) 10203, (c) 4, (d) 36 × 10^4^ Hz/s. Both types of emboli generated similar low- and high-frequency modulation.

To test the FM method, it was necessary to ensure that a similar pressure level from the transducer was used as in Girault et al. (2011a). A pressure level of 400 kPa using their commercial system generated an adequate radiation force to discriminate between small gaseous and solid emboli. With the Doppler-Box settings used in this experiment (PRF = 5 kHz, N = 25 cycles, f = 2.0 MHz, I_SPTA_ = 480 mW/cm^2^), a pressure level of ∼480 kPa was estimated. To quantify the frequency modulation present in the Doppler signal, a frequency modulation index (FMI) was calculated in Hz/s. The embolic signal was viewed in a frequency-time domain using a Wigner-Ville transform (Fig. 3). To quantify the FMI, a similar method to Girault et al. (2011b) was used:

(2) $$FMI=\frac{{\text{Δ}}_{f}\left(t\right)}{{\text{Δ}}_{t}}=\frac{F_{D}\left(t-{\text{Δ}}_{t}/2\right)F_{D}\left(t-{\text{Δ}}_{t}/2\right)}{\text{Δ}t}$$

where Δ_t_ is the temporal interval in which the modulation is significant. The maximum amplitude of the signal was located and Δ_t_ was determined by locating points on either side of this maximum that had an amplitude >70% of the maximum value_._ FMI was then calculated using Equation 2 (*i.e.,* the slope of the signal in the Wigner-Ville display representing the rate of change of the Doppler frequency was computed). If the signal exhibited complex modulation, the user could manually input the start and endpoints of the frequency modulation in the signal to calculate the FMI as described by Girault et al. (2011b). Figure 3 displays typical Doppler embolic signals from plaque and bubbles. Absolute FMI values were used for analysis, where highly negative and highly positive values both indicate strong modulation.

Six other embolic signal properties were also analyzed, along with FMI, to establish whether any of these properties differed between solid and gaseous emboli. These included PEBR (dB), mean embolus/background ratio (MEBR, dB), embolic signal duration (d_emb_, ms), Doppler shifted embolic frequency (f_D_, Hz), embolic velocity (v, m/s) and effective sample volume length (SVL_eff_, cm). MEBR was calculated by replacing I_PE_ in Equation 1 with the average backscattered signal intensity, I_E_ (see Fig. 2). This is the integrated backscattered power from the embolic signal in flowing blood with respect to time and incorporates fluctuations in the backscattered power as the embolus passes through the sample volume. MEBR is generally 5–9 dB less than PEBR. Embolic signal duration was defined as the period when the amplitude of the backscattered signal was 5 dB above the background signal. The Doppler shifted frequency, f_D,_ was determined using

(3) $$f_{D}=\frac{no.\;of\;complete\;half\;cycles}{2.d_{emb}}$$

Embolic velocity was calculated from the Doppler equation:

(4) $$v=\frac{f_{D}c}{2f_{i}\;\cos\;\vartheta }$$

where f_D_ is Doppler frequency, c is the speed of sound in soft tissue (1540 m/s), f_t_ is the transducer frequency (2 MHz) and θ is the angle of insonation (40 degrees). The effective SVL, SVL_eff_, was calculated as the product of signal duration and embolus velocity:

(5) $$SVL_{eff}=d_{emb}\cdot v$$

The difference between peak and mean EBR, ΔEBR = PEBR – MEBR, was also studied to determine if this quantity could help differentiate between solid and gaseous emboli. This estimates the difference in the maximum backscattered intensity from the embolus as it travels through the sample volume compared with the averaged backscattered intensity. Because a pointlike spherical embolus will backscatter ultrasound more consistently than a non-Rayleigh scattering, irregularly shaped particle, the difference between PEBR and MEBR is expected to be less for spherical bubbles than solids. Therefore we expect that a greater difference in ΔEBR will be obtained for irregularly shaped emboli, such as the solid particles used in this study, compared with spherical point-like gaseous bubbles.

### Statistics

Continuous variables are displayed as mean ± standard deviation for normal distributions or median and 95% confidence intervals (CI) if non-normally distributed. A one-sample Kolmogorow-Smirnov test was used to check for normality, and comparison of normal and non-normal distributions performed using either a Student's *t*-test or Mann-Whitney U test, as appropriate. Statistical significance was assumed at a level of *p* = 0.05. Receiver operating characteristic (ROC) curves were used to evaluate sensitivity and specificity and to determine the optimum threshold for successful detection of solid emboli.

## Results

**Fig. 4:**
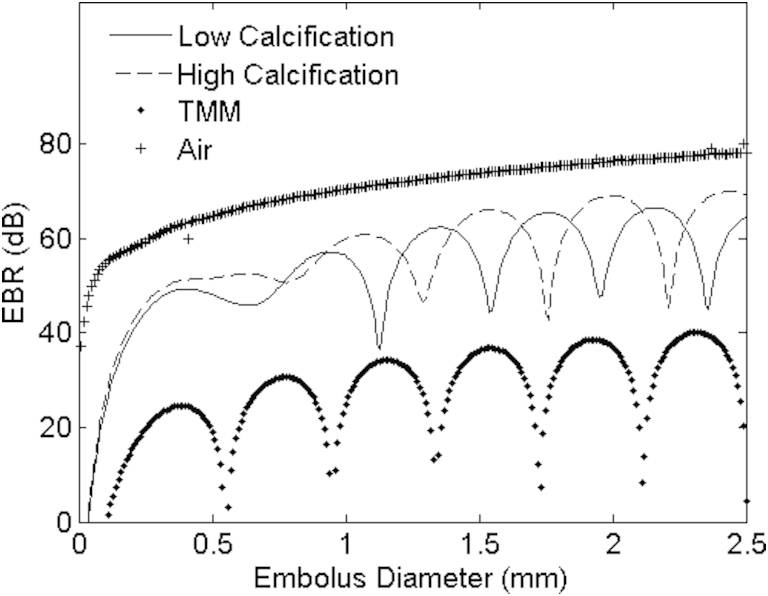
Theoretical curves as described in Moehring and Klepper (1994) for 2 MHz insonation frequency for predicted embolus/background ratio (EBR) values with embolus diameter for plaque (with high and low calcification), tissue-mimicking material (TMM) to imitate blood clots and air in a blood-mimicking fluid (BMF). Multiple solutions for embolus type and diameter exist for a range of EBR values. For plaque, different compositions will have different acoustic properties thus producing alternative theoretical curves. This makes sizing of solid particles based on EBR values alone extremely difficult.

To test the DF and FM technique, Doppler embolic signals were recorded from 355 bubbles and 67 solid emboli (42 pieces of plaque and 25 pieces of TMM). These plaque and TMM pieces were repeatedly injected into the phantom under pulsatile flow conditions. Forty-two pieces of plaque with approximate diameters ranging between 0.3 and 1.55 mm were cut from the excised carotid plaque. Histologic examination indicated that the plaque was calcified (grade 2) with a large lipid core and was probably unstable. No thrombus or fibrous tissue was found. The 25 pieces of TMM had approximate diameters ranging between 0.5 and 2.0 mm. Based on their backscattered signal intensities, modeled for bubbles circulating in a weakly diluted BMF solution, bubble diameters ranged from 0.01 to 2.5 mm (Fig. 4); the theoretical model is described elsewhere in Moehring and Klepper (1994). The same emboli were used to simultaneously assess both DF and FM techniques under pulsatile flow.

To test how the FM method performed for the characterization of larger emboli under steady flow, 61 Doppler signals were also recorded from a different set of TMM particles (15 particles, with diameters between 0.5 and 2 mm) and 137 bubbles (diameter range: 0.02–1.77 mm).

### Automatic detection and dual-frequency technique

The performance of the automated detection software was assessed for 15 TMM particles (0.5–2 mm) and ambient bubbles (0.01–2.5 mm diameter). The TMM particles were repeatedly injected (10 times) to the flow phantom under pulsatile flow conditions. A total of 81 emboli were detected through review of the recordings by a human expert (gold standard). Some of the recordings were rejected because of multiple emboli traveling through the sample volume at the same time. This made individual analysis impossible. From the 81 detected emboli, 6 of these were not detected by the DF system because their EBR values fell below the 9 dB default threshold assumed by the software for DF embolus detection. Of the remaining 75 emboli, the software misclassified 4 artifacts as emboli and 15 emboli as artifacts. Approximately 60% of the misclassified emboli had peak EBR values of 10 dB, just slightly above the detection threshold. The remaining 40% had peak EBR values >45 dB (average of ∼69 dB). From these data, the sensitivity and specificity of the automated embolus detection software in distinguishing emboli (particles and bubbles) from artifacts was 80% and 99%, respectively.

Classification of solid and gaseous emboli using the dual-frequency technique under pulsatile flow was then assessed using 355 signals from bubbles and 345 signals from solid emboli (141 signals from TMM and 204 signals from plaque). Of these, 4% of plaque, 7% of TMM and 10% of bubbles were wrongly labeled as artifacts. The majority of pieces of plaque and TMM were incorrectly classified as gaseous, with 18% sensitivity and 89% specificity for correct classification of solid emboli based on the remaining “successfully” detected emboli.

### Frequency modulation method

**Fig. 5:**
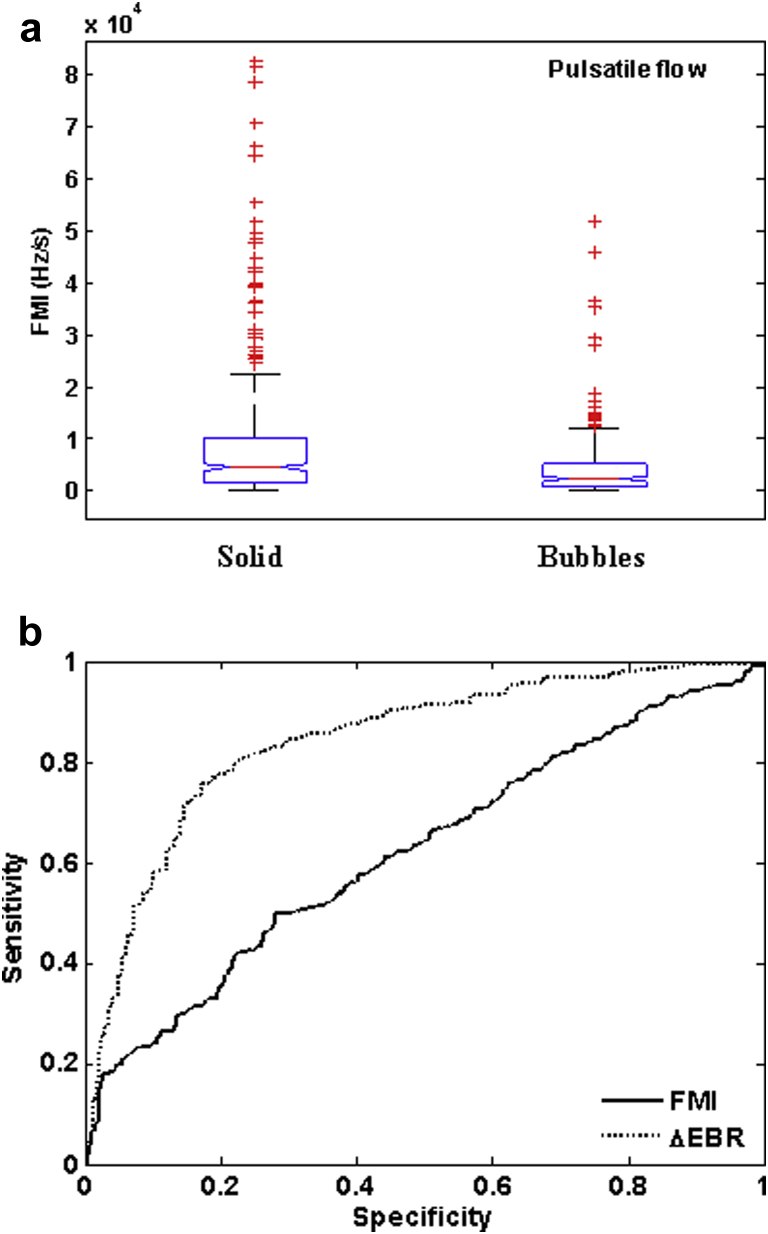
(a) Whisker plot showing frequency modulation indix (FMI) values for solid (tissue-mimicking material [TMM] and plaque) and bubbles for pulsatile flow. Although there was a statistically significant difference between the medians (*p* = 3 × 10^−9^, Mann-Whitney), the large overlap in distributions between bubbles and solid emboli means that it would be unreliable to categorize emboli based on FMIs alone. Notches display the 95% confidence interval endpoints. *Red crosses* are individual data points. (b) Receiver operating characteristic (ROC) curve for ΔEBR and FMI for all solid emboli. ΔEBR produces a higher sensitivity and specificity (77%, 81%) compared with FMI (50%, 72%).

The same Doppler embolic signals used to test the dual-frequency technique were then used to assess the FM method. Histograms of absolute FMI values for both bubbles and solid emboli suggested a non-normal distribution for FMI values in pulsatile flow, which was confirmed using a one-sample Kolmogorow-Smirnov test. Figure 5a displays the median and inter-quartile values for each embolus type in pulsatile flow. Notches display the 95% confidence interval (CI) endpoints. The median (and 95% CI) absolute FMI value was 4.8 (0.28, 44.5) kHz/s for plaque, 3.8 (0.30, 28.7) kHz/s for TMM and 2.3 (0.21, 13.7) kHz/s for bubbles. Because of large variances, no statistical significance was observed between absolute FMI values for TMM and plaque (*p* = 0.12, Mann-Whitney). By grouping TMM and plaque together to form a “solid” group, a statistical significance was observed between bubbles and solid particles (*p* = 3 × 10^-9^, Mann-Whitney). The outliers in the bubble data set (5%) were all ≤100 μm in diameter, but because this is such a small percentage, no conclusions can be drawn between bubble size and very high frequency modulation (>10 kHz/s). For the solid emboli, the range in PEBR values for the outliers (11%) was between 24 and 52 dB. Based on theoretical predictions, this PEBR range encompasses the size range used in the study (0.5–2.0 mm), so no direct link between size and the spread in FMI values can be made.

ROC curves for absolute FMI values were compiled for the two types of solid emboli separately and then for both combined (Table 2 and Fig. 5b). The FM method performed poorly for distinguishing between TMM and bubbles, giving a sensitivity and specificity of 57% and 59%, respectively, for correct identification of TMM. The FM method performed slightly better when differentiating between plaque particles and bubbles (sensitivity: 72%; specificity: 50%).

**Table 2:** Specificity and sensitivity for detecting solid emboli using the frequency modulation (FM) method, the dual-frequency (DF) technique and ΔEBR = peak embolus/background ratio (PEBR) - mean EBR (MEBR) for pulsatile flow. Non-pulsatile flow results for tissue-mimicking material (TMM) displayed in parentheses

Embolus type	Sensitivity (%)	Specificity (%)
Frequency modulation method		
TMM	57 (52)	59 (72)
Plaque	72	51
All solid	72	50
Dual-frequency technique		
TMM	14	89
Plaque	20	89
All solid	18	89
ΔEBR		
TMM	75 (58)	80 (79)
Plaque	79	83
All solid	77	81

In non-pulsatile flow, a non-normal distribution was also confirmed for absolute FMI values using a one-sample Kolmogorow-Smirnov test. Median FMI values were 17.4 (95% CI: 0.62, 83.1) kHz/s for TMM and 11.0 (95% CI: 1.35, 35.6) kHz/s for bubbles. A statistically significant difference was observed between bubbles and TMM in non-pulsatile flow (*p* = 0.01, Mann-Whitney). Absolute FMI values gave a sensitivity and specificity of 52% and 72%, respectively, in non-pulsatile flow. This was a slight improvement in specificity compared with pulsatile flow (sensitivity: 57%; specificity: 59%).

**Fig. 6:**
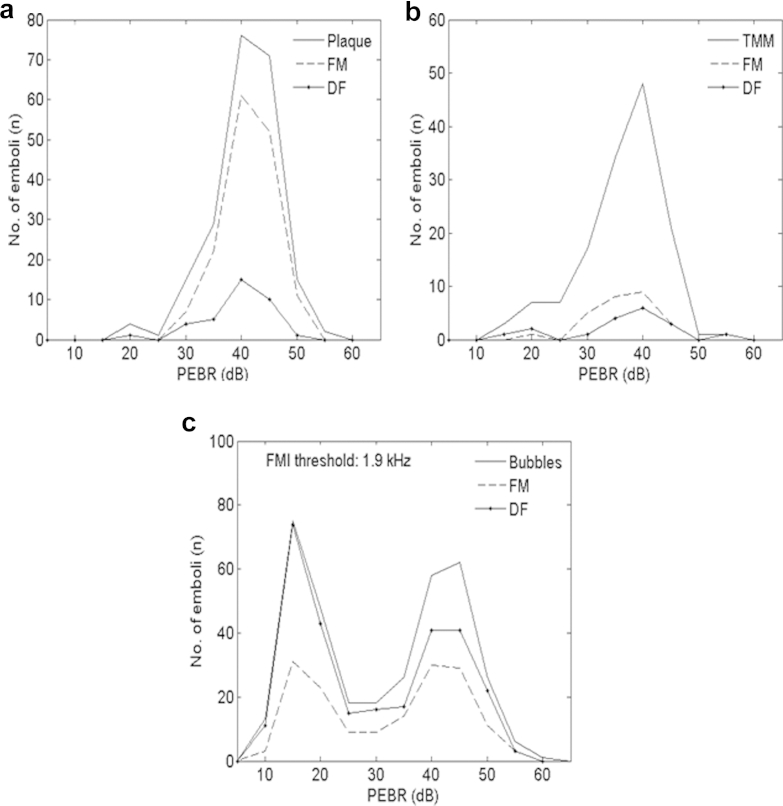
The number of emboli correctly identified as solid or gaseous for plaque, tissue-mimicking material (TMM) and bubbles using the dual-frequency (DF) technique and frequency modulation (FM) method. *Solid line* is the known distribution of peak embolus/background ratio (PEBR) for emboli. (a) The FM method outperforms the DF technique for detecting plaque particles. (b) Neither technique performs adequately to detect tissue-mimicking material (TMM) particles. (c) Comparison of both techniques for detecting gaseous emboli with plaque particles present. The DF technique proves highly sensitive to small bubbles < 25 dB PEBR. The 1.9 kHz threshold was found using receiver operating curve (ROC) analysis and gives the highest sensitivity and specificity for detecting gaseous emboli.

Although scattering theory predicts a non-linear relationship between embolus diameter and EBR, larger solid particles generally generated higher EBR values that were more likely to be confused with gas bubbles (see Fig. 4). To assess whether performance of the DF and FM system was affected by backscattered Doppler intensity, a comparison of detection rates for PEBR values is presented in Figure 6. It can be seen that the FM method correctly classified a higher proportion of high-intensity signals as plaque particles than the DF method. However, the DF technique surpassed the FM method for detecting small bubbles (<50 μm) in the 10–25 dB PEBR range (see Fig. 6c). The FM threshold of 1.9 kHz quoted in Figure 6c was found from ROC analysis (*i.e.,* this value gave the highest sensitivity and specificity for detecting gaseous emboli).

### Doppler embolic signal properties

Histograms were calculated for the six other embolic signal properties. Because data were non-normally distributed, a Mann-Whitney U test was applied to identify any significant differences between embolus properties for the three different embolus compositions. Median and 95% confidence intervals are given for each signal property in Table 3 (values are provided for both pulsatile and non-pulsatile flow for TMM and bubbles).

**Table 3:** Median and 95% confidence intervals of embolic signal properties for plaque, tissue-mimicking material (TMM) and bubbles in pulsatile and non-pulsatile flow

Doppler signal property	Plaque	TMM		Bubbles	
	Pulsatile median (95% CI)	Pulsatile median (95% CI)	Non-pulsatile median (95% CI)	Pulsatile median (95% CI)	Non-pulsatile median (95% CI)
d_emb_ (ms)	32 (20, 86)	38 (19, 77)	18 (13, 28)	26 (13, 80)	21 (15, 27)
PEBR (dB)	44 (33, 51)	40 (23, 48)	41 (31, 64)	37 (16, 54)	62 (45, 69)
MEBR (dB)	34 (24, 41)	30 (16, 39)	31 (22, 54)	30 (9, 46)	53 (37, 60)
f_D_ (Hz)	664 (336, 1029)	559 (303, 918)	1098 (802, 1399)	584 (312, 1070)	1313 (976, 1593)
v (cm/s)	30 (15, 46)	25 (14, 41)	49 (36, 62)	26 (14, 48)	58 (43, 71)
SVL_eff_ (cm)	1.0 (0.6, 1.5)	1.0 (0.6, 1.4)	0.8 (0.6, 1.5)	0.8 (0.3, 1.4)	1.3 (0.8, 1.5)
FMI (kHz)	4.8 (0.3, 44.5)	3.8 (0.3, 28.7)	17.4 (0.6, 83.1)	2.3 (0.2, 13.7)	11.0 (1.4, 35.6)
ΔEBR (dB)	9.5 (8.7, 10.4)	9.5 (8.5, 10.6)	9.5 (8.5, 10.1)	7.4 (6.3, 8.22)	8.9 (8.4, 9.2)

CI = confidence interval; d_emb_ = embolic signal duration; f_D_ = Doppler shifted frequency; FMI = frequency modulation index; MEBR = mean embolus/background ratio; PEBR = peak embolus/background ratio; SVL_eff_ = effective sample volume length; v = embolic velocity.

#### Pulsatile flow

As expected for two different populations of signals, a statistically significant difference was observed (*p* < 0.05) between the median values of all eight embolic signal properties (including FMI) when comparing plaque particles with bubbles. However, a large overlap in distributions was noted, that makes it impossible to confidently predict the composition of any individual signal. For TMM particles, the median MEBR value, f_D_, and *v* were not statistically significant when comparing TMM with bubbles. With the remaining signal properties, despite a statistically significant difference in median values, a large overlap in distributions was also observed.

ROC curves were compiled for TMM and plaque separately and with both combined for each Doppler signal property to evaluate the sensitivity and specificity for detection of solid emboli. All but one of the eight signal properties studied gave a combined sensitivity and specificity > 65%. From ROC curve analysis, a sensitivity of 77% and specificity of 81% was achieved to detect all solid emboli using ΔEBR (see Fig. 5b). There was a marginal improvement for detecting plaque emboli (sensitivity: 79%; specificity: 83%) and a slight decrease for detecting TMM (sensitivity: 75%; specificity: 80%) when analyzed separately.

#### Steady flow

As expected for two different populations of signals, all seven embolic signal properties generated statistically significant differences between their median values when comparing TMM and bubbles in steady flow. Using ROC curves, MEBR gave the highest sensitivity and specificity of 83% and 94%, respectively, compared with the other properties. Unlike pulsatile flow, the sensitivity and specificity for detecting TMM using ΔEBR was worse with 58% and 79%, respectively.

## Discussion

The aim of this study was to directly compare FM and commercially available DF embolus classification techniques for discriminating between large pieces of solid debris and bubbles. An accurate differentiation technique for determining embolus composition would be useful for providing clinicians with diagnostic information for clinical decision making. Although both techniques previously have been reported as having promising results for classification of microemboli (<100 μm) *in vitro* (Girault et al., 2011a, Russell and Brucher, 2002, Russell and Brucher, 2005), based on our results, neither technique performed adequately in distinguishing between large solid particles with similar EBR values to gas bubbles.

The commercial dual-frequency software performed well for automated detection of emboli, achieving 80% sensitivity and 99% specificity. One in five emboli were misclassified as artifacts, mainly comprising embolic signals with intensities greater than 65 dB or less than 10 dB. Although bubble signals <10 dB are unlikely to be clinically significant, the risk of ignoring solid emboli with intensities <10 dB or >65 dB corresponding to large pieces of thrombus or plaque debris may prove detrimental to the patient if the current software was relied on for clinical input. In clinical settings where low-intensity embolic signals associated with thrombus are expected to occur (*e.g.,* after carotid endarterectomy), it remains advisable to use a trained human expert for embolus detection because small pieces of thrombus are known to generate embolic signal intensities several dB below the threshold for automated embolus detection (Chung et al. 2006). For signals > 65 dB, it is possible that signal saturation occurred and the dynamic range of the system is not sufficient to detect intensities around this value. The automatic software therefore misclassifies these high-intensity emboli because the signal appears to occur at more than one depth.

Although the DF technique performed well at embolus detection, classification of emboli as solid or gaseous was extremely inaccurate (18% sensitivity, 89% specificity). Four out of five pieces of plaque and TMM introduced to our phantom were misclassified as gaseous. Considering that our *in vitro* experiment was performed in the absence of clinical factors such as patient movement, or incomplete insonation of the vessel, these poor results confirm that the dual-frequency technique is not accurate enough for use in clinical or research studies.

The FM method performed better than the DF technique in discriminating solid emboli from bubbles (see Table 2); however, a large overlap in the distribution of FMI values between solid and gas bubbles means that it would be unreliable to categorize emboli based on FMI alone (see Fig. 5). Figure 6 (a and b) compares DF and FM sensitivity for correct classification of plaque and TMM as a function of PEBR. Figure 6c shows the same information for correct classification of bubbles. The FM method was more successful at correctly classifying plaque debris as solid than the artificial thrombus mimic and was consistently superior to the DF technique in the detection of solid particles. Interestingly, the DF technique was highly sensitive in detecting small gas bubbles (<50 μm, PEBR 10–25 dB) (see Fig. 6c). This correlates with the previous results of Russell and Brucher, 2002, Russell and Brucher, 2005 in their *in vitro* experiment, where the DF technique correctly classified 94.3% of gas bubbles with diameters in the range of 8–25 μm.

Unfortunately, we were unable to reproduce the promising experimental results of Girault et al. (2011a). In this study large solid particles introduced to our phantom were associated with higher median FMI values than bubbles. The range of FMI values was broad for both bubbles (11.6–5.4 × 10^4^ Hz/s) and plaque (2.3–10.8 × 10^4^ Hz/s) compared with those quoted in Girault et al. (2011a) for pork particles (<10^2^ Hz/s) without skull present. Although our results appear to be at odds with Girault et al.’s previous findings, the FM changes detected in Girault et al.'s study were generated by much smaller bubbles, which were deflected under steady flow conditions. Our experiment features physiologically realistic pulsatile flow, which has potential to exert uneven forces on irregularly shaped large particles and induce additional motion. When Girault et al. (2011b) studied different velocity and pressure distributions, it was found that both parameters had a significant impact on the frequency modulation and displacement of the embolus. It was concluded that pulsatile flow adds an additional acceleration that complicates embolus trajectory and makes predictions of displacement based on frequency modulation difficult. If this is the case, then the accuracy of the FM method will be reduced for large, irregularly shaped particles moving in pulsatile flow.

Seven other embolic signal properties were also analyzed for each individual embolus. All of these properties have been analyzed in previous studies (Droste et al., 1994, Georgiadis et al., 1994, Grosset et al., 1993, Markus et al., 1993, Smith et al., 1997, Smith et al., 1998). PEBR, MEBR and ΔEBR represent the backscattered signal intensity from the embolus. From Figure 4 one can see that gaseous emboli backscatter at much greater intensities compared with solid emboli in a blood-filled vessel. This is due to the large impedance mismatch between air and blood. However, from ROC curve analysis, none of these properties produced an acceptable sensitivity and specificity (>90%) for detection of solid emboli in a clinical setting. ROC curve analysis for ΔEBR gave an improved result for solid emboli (TMM and plaque) in pulsatile flow (sensitivity: 77%; specificity: 81%), which is similar to that found using more sophisticated expert system models (Darbellay et al., 2004, Devuyst et al., 2001).

Although this *in vitro* study was designed to examine whether the FM method and the dual-frequency technique could be used to differentiate between macrobubbles and large solid emboli, certain limitations may have affected the outcome of our results. It is possible that the injection of particles into the flow phantom provided an extra acceleration that may have masked the radiation force effect caused by the ultrasound beam. To minimize this error the injection port was placed ∼0.5 m from the Doppler sample volume.

Skull, which has been shown to cause additional attenuation and distortion of the ultrasound beam, was not used in this experiment. Evans and Gittins (2005) found that the errors in EBR ratios with and without bone were similar and therefore we would expect that the addition of bone would have made little difference to our results. In clinical situations, variations between the thicknesses of skull at the temporal windows of patients will result in different attenuation of the ultrasound beam, which in turn will affect the ultrasound beam pressure. This in turn suggests a patient-specific threshold may be needed to optimize the FM technique, so we would therefore expect that the levels of sensitivity and specificity reported here are likely to be worse in the presence of the skull.

Angell and Evans (2003) reported that measured values of absolute EBR can vary between 4 and 12 dB depending on sample volume shape and beam–vessel misalignment; however, measurements that depend on ratios of EBR values such as peak/mean EBR and EBR measured at two different frequencies are largely immune to these errors.

Using a highly diluted BMF artificially increases EBR values, and this has been taken into account in the modeling. Although the scattering cross section for BMF is similar to that for blood because of their similar acoustic properties, the number density of scatterers is greatly reduced and does not reflect typical hematocrit values for blood. However, because PEBR and MEBR are calculated as a ratio based on the backscattered intensity of the embolus in blood through I_PE+B_ and I_E+B_ respectively divided by the reduced backscattered intensity from the weak BMF, this effective lowering of hematocrit has been compensated for by our model.

A limitation of the plaque emboli used in this study is that they were made from the excised plaque of just one patient undergoing carotid endarterectomy; therefore it may be that different results would be found for different plaque compositions. It is known that embolism from calcified atherosclerotic plaque can occur during cannulization of the aortic arch for cardiopulmonary bypass. Indeed plaque emboli with surface areas between 0–82.5 mm^2^ have been reported during cardiac surgery (Banbury et al. 2003). It would be interesting to see if a thrombotic plaque behaved in a similar manner to the calcified plaque used in this experiment. Thrombotic plaque has a lower density (1190 kg/m^3^) compared with calcified plaque (1450 kg/m^3^) (Rahdert et al. 1999) and so may experience a higher ARF, leading to higher FMI values.

The solid emboli used in this study had irregular surfaces, and the theoretical predictions in Figure 4 are modeled on a spherical embolus. It is important to acknowledge that predicted EBR values may be affected by plaque shape, which has not been included in our model. It should also be acknowledged that bubbles of 3 μm in diameter are naturally resonant at 2 MHz and will backscatter at a much higher intensity (∼40 dB) compared with similar sized bubbles (1–5 μm). From Figure 4, this value is comparable to backscatter from a larger sized bubble (∼18 μm) or a solid embolus composed of plaque (0.2–1.1 mm). Although bubbles within a 3–18 μm diameter range will dissolve quickly in blood and pose low risk to the patient (Branger and Eckmann 1999), a solid embolus of 0.2–1.1 mm diameter is clinically significant. Therefore in clinical situations where patients are at risk to a mixture of both bubbles and plaque emboli, such as during cardiac surgery, distinguishing emboli based on their backscattered intensities alone is not advised.

## Conclusions

In this study, both the dual-frequency technique and FM embolus discrimination methods were unable to reliably differentiate large pieces of plaque and thrombus-mimicking material from bubbles. The 18% sensitivity and 89% specificity associated with commercial dual-frequency techniques for detection of solid macroemboli are likely to give highly misleading results in clinical use. Although the presence of frequency modulation in the Doppler signal was a better predictor of embolus composition (plaque: 72% sensitivity, 50% specificity; TMM: 57% sensitivity, 59% specificity), the accuracy of this type of analysis is heavily reduced for large pieces of debris circulating in pulsatile flow conditions and the low specificity is likely to generate high numbers of false positives results, especially in clinical settings where a high proportion of bubbles are present (*e.g.,* during cardiac surgery). In pulsatile flow, the difference in PEBR and MEBR (ΔEBR) appears to provide the optimum means of differentiating large pieces of solid debris from gas bubbles. This yields 77% sensitivity and 81% specificity, which is better than either the DF or FM technique.
